# Processing and Characterization of Bioplastics from the Invasive Seaweed *Rugulopteryx okamurae*

**DOI:** 10.3390/polym14020355

**Published:** 2022-01-17

**Authors:** Ismael Santana, Manuel Félix, Antonio Guerrero, Carlos Bengoechea

**Affiliations:** Higher Polytechnic School, University of Seville, Calle Virgen de África, 7, 41011 Sevilla, Spain; isantana@us.es (I.S.); mfelix@us.es (M.F.); aguerrero@us.es (A.G.)

**Keywords:** *Rugulopteryx okamurae*, bioplastics, DMA, injection molding, seaweed

## Abstract

The seaweed *Rugulopteryx okamurae,* from the Pacific Ocean, is considered an invasive species in the Mediterranean Sea. In this work, the use of this seaweed is proposed for the development of bio-based plastic materials (bioplastics) as a possible solution to the pollution produced by the plastic industry. The raw seaweed *Rugulopteryx okamurae* was firstly blended with glycerol (ratios: 50/50, 60/40 and 70/30), and subsequently, they were processed by injection molding at a mold temperature of 90, 120 and 150 °C. The rheological properties (frequency sweep tests and temperature ramp tests) were obtained for blends before and after processing by injection molding. The functional properties of the bioplastics were determined by the water uptake capacity (WUC) values and further scanning electron microscopy (SEM). The results obtained indicated that E’ was always greater than E”, which implies a predominantly elastic behavior. The 70/30 ratio presents higher values for both the viscoelastic moduli and tensile properties than the rest of the systems (186.53 ± 22.80 MPa and 2.61 ± 0.51 MPa, respectively). The WUC decreased with the increase in seaweed in the mixture, ranging from 262% for the 50/50 ratio to 181% for the 70/30 ratio. When carrying out the study on molded bioplastic 70/30 at different temperatures, the seaweed content did not exert a remarkable influence on the final properties of the bioplastics obtained. Thus, this invasive species could be used as raw material for the manufacture of environmentally friendly materials processed by injection molding, with several applications such as food packaging, control–release, etc.

## 1. Introduction

*Rugulopteryx okamurae* (RO), also known as *Dictyota marginata*, *Dilophus marginatus*, or *Dictyota okamurae* [1,2], is a species of brown algae belonging to the *Dictyotaceae* family, originally from the coasts of the warm and temperate northwestern Pacific Ocean (Korea, Japan, China, Taiwan and the Philippines) [3]. This alga has been introduced from the Pacific Ocean to the Mediterranean through the Strait of Gibraltar. The Spanish Mediterranean coasts and those of the Strait of Gibraltar present a highly favorable environment for the species, favoring its expansion and an increase in the derived impacts. Consequently, on 1 December 2020 it was included in the Spanish Catalog of Invasive Exotic Species since it represents one of the main threats to biodiversity in the Mediterranean, due to its ability to spread and adapt [4]. 

In 2020, García-Gómez et al. [5] reported that more than 3,000,000 m^2^ of the seabed of the Strait Natural Park was occupied by this invasive seaweed at different depths. The highest proportion of coverage occurs between 5 and 30 m, reaching 85–96%, while the smallest was found at greater depths or practically on the shores, 30–40 or 0–5 m (around 42–45%). This invasive species not only has effects on the seagrass, but it should also be noted that 5000 tons of this Asian seaweed were dislodged from the beaches of Ceuta in 2015, and 400 tons from the beaches of Tarifa only in July 2020 [6].

The great technological development from the twentieth century to the present has caused an increase in environmental pollution. Among these advances, the industrial production of non-biodegradable plastic materials stands out [7]. At the beginning of the 21st century, the average consumption of plastic was 15 kg/year per person [8]. This also accounts for a large part of the pollution that is produced, both by the emission of greenhouse gases during their manufacture and disposal, as well as by their accumulation in landfills and a high presence in the oceans due to their low biodegradability [9]. Since the 1950s, more than 7800 million metric tons have been produced, of which more than 10% ends up in the oceans once disposed. This produces worrying impacts on the seabed, its biodiversity and people due to the consumption of fish contaminated with microplastics [10]. Thus, an annual consumption of 39,000 to 52,000 microplastic particles is estimated that can negatively affect health [11].

Nowadays, an important part of this development is inconceivable without taking care of the environment, and that is why there are increasingly more restrictive regulations and a greater and growing interest in finding biodegradable materials with properties similar to those of conventional plastics. The solution of conventional plastics is the use of bioplastics, which can be defined as those plastics made from a renewable source or those which are biodegradable [12,13]. This demand for bio-based raw materials will demand a huge amount of resources, which could be supplied by both agri-food wastes or invasive species with no applications in the local market. Underutilized invasive species would provide adequate biomass for processing, which would reduce their environmental impact. The present work proposes the use of the invasive seaweed RO from the bay of Algeciras (located in the Strait of Gibraltar) as a raw material to produce bioplastics. The use of the invasive seaweed RO to generate biodegradable materials would serve both to reduce the impact of algae in the Mediterranean biodiversity and find an alternative to fossil-based plastics that pollute the planet. Depending on their properties, algae are used for various applications, such as in cosmetic or bioplastic packaging industries [14].

One of the problems when replacing conventional plastics with bioplastics are the poorer mechanical properties of the latter during their processing and end use, which can limit their potential. As a solution, a plasticizer is added to improve its mechanical properties. According to IUPAC (International Union of Pure and Applied Chemistry), a plasticizer is defined as “a substance or material incorporated into another material to improve its flexibility, compliance or viability” [15]. Plasticizers generally have a high boiling point and carbon chains of between 14 and 40 carbons, with a molecular weight of between 300 and 600 [16]. Thanks to these characteristics, plasticizers can be inserted into the structure of the polymers that make up plastics, reorganizing their three-dimensional structure, reducing the intramolecular forces of the polymer and allowing its mobility [17]. Due to this function, the choice of plasticizer for the plastic to be developed is important, as it will affect its final properties. Other parameters to consider when choosing a plasticizer are the boiling or melting temperature, polarity or solvation. Moreover, little or no toxicity, such as that of fatty acid esters or vegetable glycerol, is desired when selecting a plasticizer [18].

The selection of the technique and conditions used when processing bioplastics is also highly influential in their final properties. It should be noticed that Mass Flow Rate (MFR) is also of extreme importance when selecting a processing technique for bioplastics. Some authors have pointed out that some biodegradable plastics, such as polylactide (PLA), experience a greater increase in MFR when increasing the processing speed than conventional plastics, such as low-density polyethylene (LDPE) [19]. This clearly affects the energy efficiency of the processing techniques. There are different techniques for processing bioplastics, such as compression molding, extrusion or injection molding, among others. Compression molding consists of two metal molds that apply a certain force on the sample to produce the bioplastic. As the sample flows into the mold, it acquires its shape [20]. Extrusion is the most widely used technique for processing polymers, and consists of a continuous process, where the polymer is transported through a barrel where it is heated to enhance its flowability through a final, conveniently shaped orifice. The polymer cools as it exits and results in a constant section solid [21]. Injection molding consists of the softening of a plastic material under the appropriate conditions and its subsequent introduction under pressure into the cavities of a mold, in which it is brought to a temperature at which the pieces can be extracted without deforming. The injection molding process can be divided into two main stages: injection and compaction. First, the substance to be molded is introduced into a tank that is heated to a certain temperature that allows the material to flow for subsequent injection into the mold, by means of a piston which exerts a certain pressure. This is typically called the injection stage. The compaction stage is the second part of the process and occurs with the sample already inside the cavities of the mold, which is heated to the molding temperature. In the case of biopolymers, the molding temperature can be higher than that of the cylinder, so as to promote crosslinking and fix the structure. During this stage, the injected homogeneous mixture is subjected to a certain stable pressure by the piston until its extraction [20]. Among all the different techniques available for dry bioplastic processing, this work selected injection molding due to its versatility and scalability.

This processing technique has been already used to manufacture bioplastics from gluten [22], soy protein [23] or blood plasma from the meat industry [24], among others. Even if different examples of the development of bioplastics have already been reported [14]. There is no such information from the invasive macroalgae RO. The qualities that macroalgae offer (low cost, low toxicity, proper mechanical properties) make them a good candidate for producing bioplastics [25].

The aim of this work has been the development and characterization of bioplastic materials based on RO seaweed. To this end, RO was firstly blended with glycerol (ratios: 50/50, 60/40 and 70/30), and subsequently, bioplastics were processed at 120 °C. Subsequently, the effect of mold temperature was also analyzed by sample processing at 90 and 150 °C (RO/GLY ratio: 70/30). The rheological properties (frequency sweep tests and temperature ramp tests) were obtained for blends before and after processing by injection molding, whereas the functional properties of the bioplastics obtained were assessed by water uptake capacity (WUC) and electron scan microscopy (SEM).

## 2. Materials and Methods

### 2.1. Materials and Sample Preparation

The seaweed RO used in the present study was gently supplied by the Andalusian Institute for Agricultural, Fisheries, Food and Organic Production Research and Training (IFAPA, Puerto Real, Spain). The RO was hand-picked from the bay of Algeciras, and subsequently freeze dried. Freeze-dried algae samples were ground in a kitchen blender (Mambo10070, CECOTEC, Valencia, Spain) at maximum speed, obtaining a flour where the diameters of most particles (~90%) were within the 10 to 100 μm range. GLY was used as plasticizer (Panreac Química S.A (Castellar del Vallès, Spain). The proximate composition of the seaweed is 18.47 ± 0.35% ashes, 13.48 ± 0.26% water, 9.76 ± 0.16% proteins (conversion factor *N*-protein: 4.92 [26]) and 11.63 ± 0.22% lipids.

Bioplastics were obtained in a two-step method: (i) RO and glycerol (GLY) was mixed in a two-blade counter-rotating batch mixer Haake Polylab QC (ThermoHaake, Karlsruhe, Germany) at room temperature and 50 rpm until system homogeneity (60, 10 and 5 min for RO/GLY ratios 50/50, 60/40 and 70/30, respectively); (ii) RO/GLY blends were injected in the lab-scale Minijet Piston Injection Molding System (ThermoHaake, Karlsruhe, Germany), obtaining 1 × 10 × 60 mm^3^ probes. The samples were processed at 60 °C (cylinder temperature), whereas the mold temperature was set at 90, 120 or 150 °C. The injection pressure was 500 bar (20 s), whereas the post-injection pressure was 200 bar (150 s). These processing conditions were similar to those from plasma porcine protein [24].

**Figure 1:**
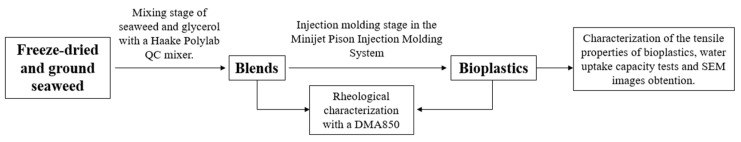
Scheme of the production process and characterization of bioplastics.

Figure 1 shows the schematic process for the processing and characterization of bioplastics.

### 2.2. Methods

#### 2.2.1. Rheological Characterization of RO/GLY Blends

##### Rheological Measurements

The RO/GLY blends obtained after the mixing stage were analyzed by dynamic mechanical analysis (DMA) in compression mode. Frequency sweep tests (from 0.1 to 10 Hz) and temperature ramp tests (from −50 to 150 °C) were performed using a DMA850 (TA Instruments, Wakefield, MA, USA). All DMA tests were performed within the linear viscoelastic region, which was determined prior to any measurement by strain sweep tests at 1 Hz. Frequency sweep tests were performed at constant temperature (25 °C), whereas temperature ramp tests were performed at a constant frequency (1 Hz). DMA tests were performed using the 15 mm diameter parallel plates geometry.

#### 2.2.2. Characterization of the RO-Based Bioplastics

##### Rheological Measurements

Final bioplastics were characterized by DMA tests with a tension clamp as geometry. For these tests, rectangular probes (10 mm × 1 mm × 15 mm) were analyzed in the DMA 850 (TA Instruments, MA, USA). Frequency sweep tests (from 0.1 to 10 Hz) and temperature ramp tests (from −30 to 180 °C) were performed using a DMA850 (TA Instruments, MA, USA). All DMA tests were performed within the linear viscoelastic region, which was determined prior to any measurement by strain sweep tests at 1 Hz. Frequency sweep tests were performed at constant temperature (25 °C), whereas temperature ramp tests were performed at a constant frequency (1 Hz). 

##### Tensile Properties

Stress–strain curves were obtained in uniaxial tensile tests until fracture using a RSA3 (TA Instruments, MA, USA). From these tests, three parameters were obtained: Young’s modulus (E), maximum stress (σ_max_) and maximum strain (ε_max_). These tests were performed using rectangular probes (60 mm × 10 mm × 1 mm) according to the standard ISO 527-2 [27]. All tensile tests were carried out at a constant elongation rate of 1 mm·min^−1^ and room temperature.

##### Water Uptake Capacity (WUC)

Water uptake capacity (WUC) was determined as follows [28]: bioplastic samples were first dried at 50 °C for 24 h and weight (w_1_); then, dried samples were immersed for 24 h in 100 mL of deionized water and weighed (w_2_). Finally, samples were lyophilized for 24 h and weighed (w_3_). WUC and soluble matter loss (SML) were calculated using Equations (1) and (2):

(1) $$\mathrm{WUC}\;\left(\%\right)=\frac{\left(w_{2}-w_{3}\right)}{w_{3}}\;\cdot 100$$

(2) $$\mathrm{SML}\;\left(\%\right)=\frac{\left(w_{1}-w_{3}\right)}{w_{1}}\cdot 100$$

##### Scanning Electron Microscopy (SEM)

Freeze-dried matrices obtained after WUC were cut into small pieces (~2.5 mm), gold coated and, finally, examined by SEM, in a ZEISS EVO (Oberkochen, Germany). A beam current of 18 pA and a working distance of 7.5–8.5 mm were employed in the microscope, with an acceleration voltage of 10 kV. Image analyses were obtained at 1000×, 500× and 200× magnification. Moreover, they were analyzed by the imaging software ImageJ (Bethesda, MD, USA) [29].

### 2.3. Statistical Analysis

All measurements were carried out at least in triplicate. The statistical analysis was carried out using the STATGRAPHICS Centurion XVIII software (The Plains, VA, USA). The standard deviation for some selected parameters was included. Significant differences (*p* < 0.05) were indicated by superscript letters.

## 3. Results and Discussion

### 3.1. Influence of the Rugulopteryx okamurae/Glycerol (RO/GLY) Ratio

#### 3.1.1. Blends

##### Mixing

**Figure 2:**
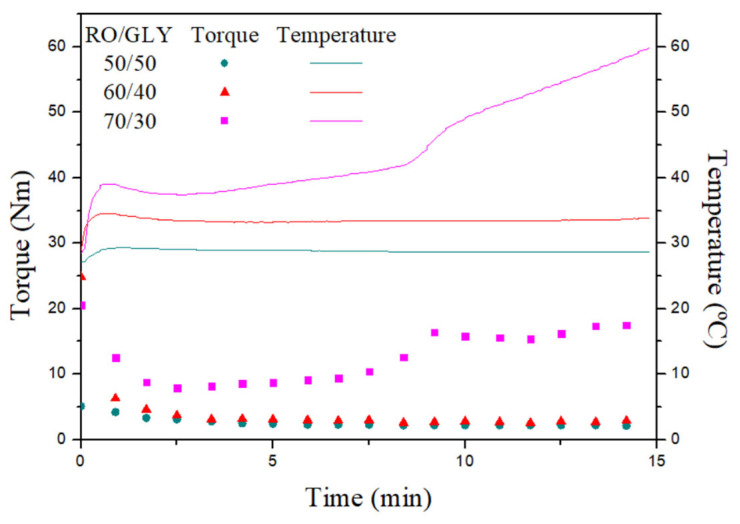
Torque and temperature values for different RO/Gly systems during the mixing stage.

After freeze drying and milling, RO seaweed was thoroughly mixed with glycerol (GLY) to obtain a homogeneous blend that was eventually injected. A picture of the resulting blends can be found in the Supplementary Material (Figure S1. Visual appearance of RO/GLY blends at different ratios (from left to right: 50/50, 60/40, 70/30)). Figure 2 shows the evolution of torque (A) and temperature (B) with mixing time for blends obtained at different RO/GLY ratios (50/50, 60/40 and 70/30). An apparent increase in both torque and temperature values was observed during the whole mixing stage as the ratio of seaweed in the blends increased, and was especially noticeable for the 70/30 system. The greater the RO/GLY ratio (i.e., the lower amount of plasticizer in the blend) resulted in a lowering of the free volume among the polymeric chains present in the seaweed biomass. As a consequence, there was greater friction which led to thermal energy dissipation that was detected as a temperature rise [22]. Moreover, 50/50 and 60/40 systems achieve a steady value for both torque (2.2, 2.8 N·m, respectively) and temperature (28.7 and 33.4 °C, respectively) after around 10 min of mixing time. At that time, both torque and temperature of the system with the greatest RO content (70/30) displayed much greater values (13.2 N·m, 43.4 °C, respectively) than the rest. Moreover, this system evolves into a sudden growth of both parameters from around 8 min, which may be related to a certain strengthening of the sample promoted by the greater proximity of the different compounds present in the biomass.

Specific mechanical energy (SME) of every system at a specific time from the mixing curves:

(3) $$\mathrm{SME}=\frac{\omega }{m}\overset{t_{\mathrm{mix}}}{\underset{0}{\int }}M\left(t\right)\delta \left(t\right)$$

where ω (in rad/s) is the mixing speed, m (in g) the sample mass, M(t) (in N·m) the torque and t_mix_ (in s) the mixing time. In order to avoid the effect of the second strengthening in 70/30, a t_mix_ of 5 min was selected for the estimation of SME shown in Table 1.

It is quite apparent that SME is greater as the seaweed content increases within the formulation, with 60/40 and 70/30 systems 1.6 and 3.9 times higher, respectively, than the system with the highest amount of plasticizer (50/50). It is well known that plasticizers ease the processability of polymeric samples and lower torque and temperatures are expected through the mixing due to a softening of the sample as their content increases [24]. 

##### Dynamic Mechanical Thermal Analysis (DMTA)

**Figure 3:**
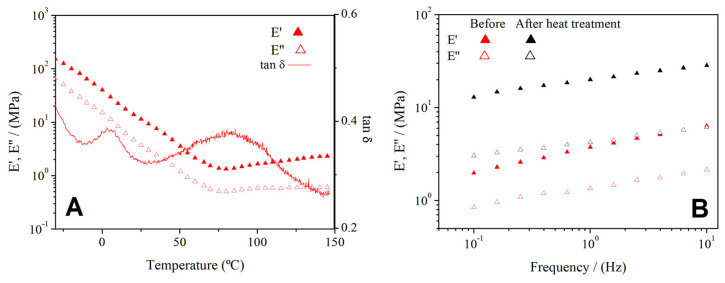
Evolution of elastic (E’) and viscous (E”) moduli with temperature (**A**) and with frequency before and after heat treatment (**B**) for the blend with a RO/GLY ratio of 60/40.

Figure 3A shows the effect of temperature from −50 to 150 °C on both viscoelastic moduli for the blend with a RO/GLY ratio of 60/40. Due to excessive flabbiness (50/50) or rigidity (70/30), blends with other RO/GLY rations did not lead to reliable experimental data. All blends softened as they were heated, which was reflected in a global decrease in both the elastic (E’) and viscous (E”) moduli when subjected to DMTA tests (data not shown). A marked decrease higher than two orders of magnitude was observed in both E’ and E”, leading to a minimum approximately at 75 °C. From then on, a slight increase and a tendency onto a steady value were observed. This behavior is qualitatively similar to that displayed by other blends including biomass from different sources such as soy, plasma porcine, among others [23,24,30]. Thermoplastic behavior generally implies a softening of the material, as reflected by the decrease in both viscoelastic moduli. This is typically associated with the fading of secondary interactions (e.g., hydrogen bonds) when heating a polymer (i.e., proteins).

When observing the evolution of the loss tangent (tan δ) along with the heating (Figure 3A), it is quite clear that in spite of the softening previously commented when heating, the sample reinforced its elastic character over its viscous one. Thus, tan δ decreased from 0.58 to 0.26. Moreover, two peaks were also observed for tan δ, one around 0 °C and the other above 82 °C, which indicate thermal transitions of the material and show the heterogeneity of the sample. As a matter of fact, when cooled down to room temperature, greater E’ and E” were obtained due to the formation of physical interactions (e.g., hydrogen bonds), as observed in Figure 3B for frequency sweep tests performed at 20 °C before and after the whole DMTA test.

Figure 3B shows the dependence of the viscoelastic moduli on frequency from 0.1 to 10 Hz before and after the heat treatment for a 60/40 RO/GLY ratio. The frequency sweep tests confirm the marked elastic character, since the elastic moduli is above the viscous moduli within the entire range of frequency studied. However, a slight dependence of the viscoelastic moduli on frequency is manifested in the E’-frequency slope, being 0.25 before the heat treatment and 0.16 after the heat treatment (Figure 3B). Despite the predominant elastic behavior, the blends obtained are injectable, since they behave similarly to other protein blends with successful injection molding results [31]. Moreover, these results also evidence the effect of temperature on the microstructure of the blends, decreasing the dependence on frequency, which reflects a lower protein chain mobility [32]. These results evidence the suitability of thermal processing methods for these blends. 

#### 3.1.2. Bioplastics

Previous blends were injection molded obtaining different bioplastic materials. A picture of the resulting bioplastics can be found in the Supplementary Material (Figure S2. Visual appearance of RO/GLY bioplastics at different ratios (from left to right: 50/50, 60/40, 70/30) molded at 120 °C). 

##### Dynamic Mechanical Thermal Analysis (DMTA)

**Figure 4:**
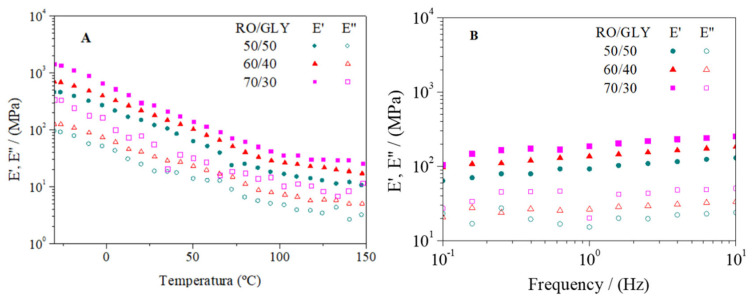
Evolution of viscoelastic moduli with temperature (**A**) and frequency (**B**) for bioplastics with different RO/GLY ratio (50/50, 60/40, 70/30).

Blends with different RO/GLY ratios (50/50, 60/40, 70/30) were subjected to injection molding at a mold temperature of 120 °C, a temperature much higher than the minimum detected in the DMTA tests of the blends (~75 °C). Figure 4 shows the DMTA tests carried out from −30 to 180 °C for the different bioplastics obtained. As observed for blends, a softening of all the bioplastics took place as they were heated, which can be associated with the disruption of the secondary structure of proteins caused by the increase in temperature. However, the decrease in the viscoelastic moduli observed in the bioplastic systems was much less pronounced than in the blends, due to the strengthening induced in the samples during the injection molding process at high temperatures and pressures. During this softening, a peak in tan δ was observed at around 80 °C for all samples, pointing out a thermal transition of these materials (data not shown). Moreover, contrary to blends, no minimum was detected during the thermal treatment which implied that no further strengthening would be expected through more exhaustive conditions. It seems that the greatest thermosetting potential was already achieved during the injection-molding processing of the seaweed-based blends. All bioplastics displayed a much more cohesive structure than the original blends and could be tested correctly, tending towards steady values at high temperatures (~125 °C). Thus, higher content in seaweed resulted in greater viscoelastic moduli, observing a growing sequence for both E’ and E” with the RO/GLY ratio (50/50 < 60/40 < 70/30) during the whole DMTA test. A similar response when increasing the biomass content in bioplastics made from proteins and GLY has already been observed [23,24]. The observed evolution of E’ and E” with RO/Gly ratio was also found in injected bioplastics from microalgae, which was attributed to the promotion of protein–protein interactions when the plasticizer content decreased [33,34]. It should be noticed that López-Rocha et al. [33] did not find the mentioned biomass content-viscoelastic properties correlation for blends, but also after the injection molding process. Samples were submitted to drastic temperature and pressure conditions when injected, which promoted the strengthening of the samples in a more efficient way than just mixing at room temperature. Therefore, when considering the value of the elastic modulus at 20 °C (E’_20_), the 60/40 system experienced an increase from around 14 (blend) to 219 (bioplastic) MPa as a result of the injection molding process. 

##### Tensile Tests

**Figure 5:**
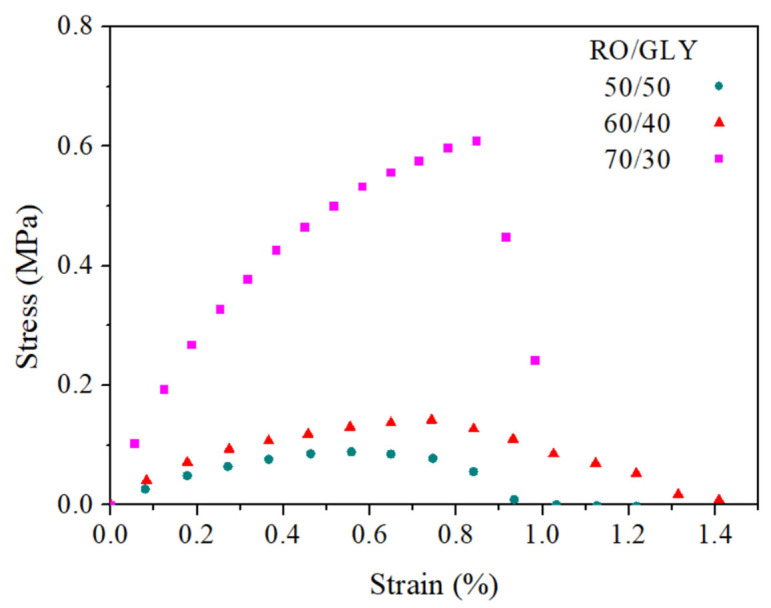
Stress–strain curves for bioplastics with different RO/GLY ratios (50/50, 60/40 and 70/30).

Tensile tests were carried out on the bioplastics with different RO/GLY ratios at constant elongation rate of 1 mm·min^−1^ to assess the effect of the biomass content on their mechanical properties. Figure 5 shows the stress–strain curves produced by the three systems studied, showing an initial linear elastic region at lower strains in which the slope corresponds to the Young’s modulus, E. When certain yield stress was exceeded, the slope began to descend as plastic deformation took place. Once the stress reached a maximum value (σ_max_), the slope decayed abruptly, especially for the 70/30 system, which implied the breakage of the probe at a maximum deformation ε_max_. In the systems studied, it is observed that as the content of seaweed in the sample increased, and the rigidity of the materials also increased, requiring higher stresses to achieve the same degree of deformation, which implies greater resistance to breakage. Moreover, this plot also showed the higher tenacity obtained for the ratio 70/30. 

Mechanical properties estimated from tensile tests for bioplastics with different RO/GLY ratios (50/50, 60/40 and 70/30) are shown in Table 2. It is observed that both E and σ_max_ increased with the content of seaweed in the formulation of the bioplastic, displaying an exponential increase that led to a remarkable increase for the 70/30 system. This evolution is in line with that observed for the viscoelastic properties of the systems obtained previously from the DMTA tests, although the evolution of E’ and E” with RO/GLY was more gradual than for the tensile properties. Regarding the ε_max_, no clear evolution could be perceived, and the 60/40 system displayed the greatest deformability. Other authors have found a similar evolution for E and σ_max_ when increasing biomass content in injection molded bioplastics [24], although they also detected an increase in ε_max_. The lower ε_max_ obtained for 70/30 should be related to its greater rigidity due to the higher strengthening achieved during injection molding, as commented before. This should be explained on basis of the lack of plasticizer, which allowed greater interaction between the polymeric chains (e.g., proteins, carbohydrates) of the material, leading to a more rigid and resistant structure. This phenomenon of ε_max_ variation has been reproduced by other authors for starch-based bioplastics. The plasticizer replaced existing intermolecular bonds in the material’s structure with hydrogen bonds, which reduces stiffness and allows flexibility, so the higher the amount of plasticizer, the greater the deformation capacity [35]. However, when the plasticizer concentration exceeds a critical value, the anti-plasticizer phenomenon can occur. In these cases, too much plasticizer in the mix can excessively weaken the cohesion between the polymer chains making it too brittle and resulting in less elongation [36].

Similar profiles are observed in other bioplastics made with proteins such as soy protein or porcine plasma protein [23,24].

##### Water Uptake Capacity (WUC)

**Figure 6:**
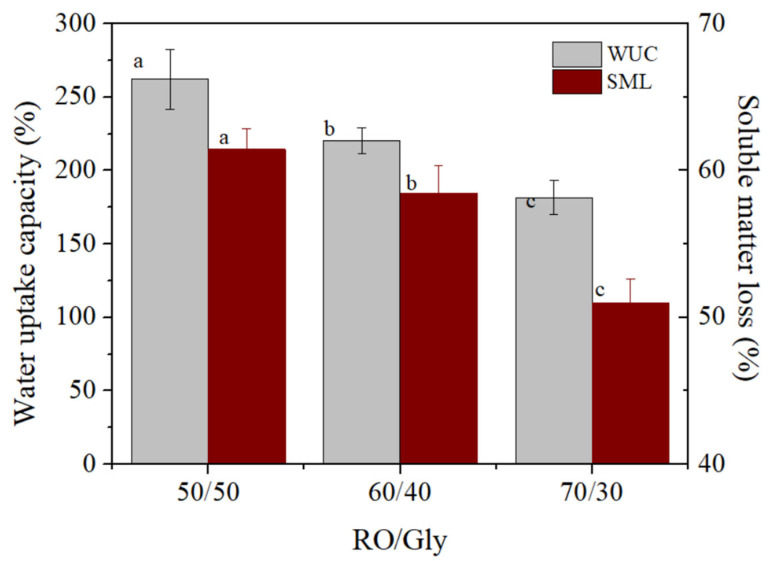
Water uptake capacity and soluble matter loss for the RO/Gly systems: 50/50, 60/40 and 70/30. Different letters (a, b, c) within the same column parameter (WUC or SML) indicate significant differences (*p* < 0.05).

Figure 6 shows the dependence of water uptake capacity (WUC) and soluble matter loss (SML) on the RO/GLY ratio of bioplastics studied. There was a significant and progressive decrease in WUC values when increasing the seaweed content in the bioplastic, going from around 260% to 180% when the RO/GLY ratio increased from 50/50 to 70/30. Similar values and evolution with biomass content were observed in bioplastics from a microalgae consortium by López Rocha et al. [33]. The greater WUC displayed by the 50/50 system can be associated with its higher glycerol content, which promotes the formation of a porous structure that may improve the absorption process, as hydrophilic GLY tends to pass onto the aqueous immersion media [23,24]. The greater rigidity and resistance measured for the 70/30 system inhibited the swelling of the bioplastic, limiting—as a consequence—its ability to absorb water. 

Regarding the SML, a tendency to decrease as the RO/GLY ratio increased was detected, being significant for the 70/30 system: both 50/50 and 60/40 bioplastics lost around 60% of mass when immersed for 24 h, while the 70/30 bioplastic lost around 50%, which is coherent with a greater number of interactions strengthening the material. Most of the SML is due to the loss of glycerol, which has a highly hydrophilic behavior [37]. However, some of the biomass (~20–30%) was also lost during immersion as the SML was always higher than the original percentage of GLY in the formulation.

##### Scanning Electron Microscopy (SEM)

**Figure 7:**
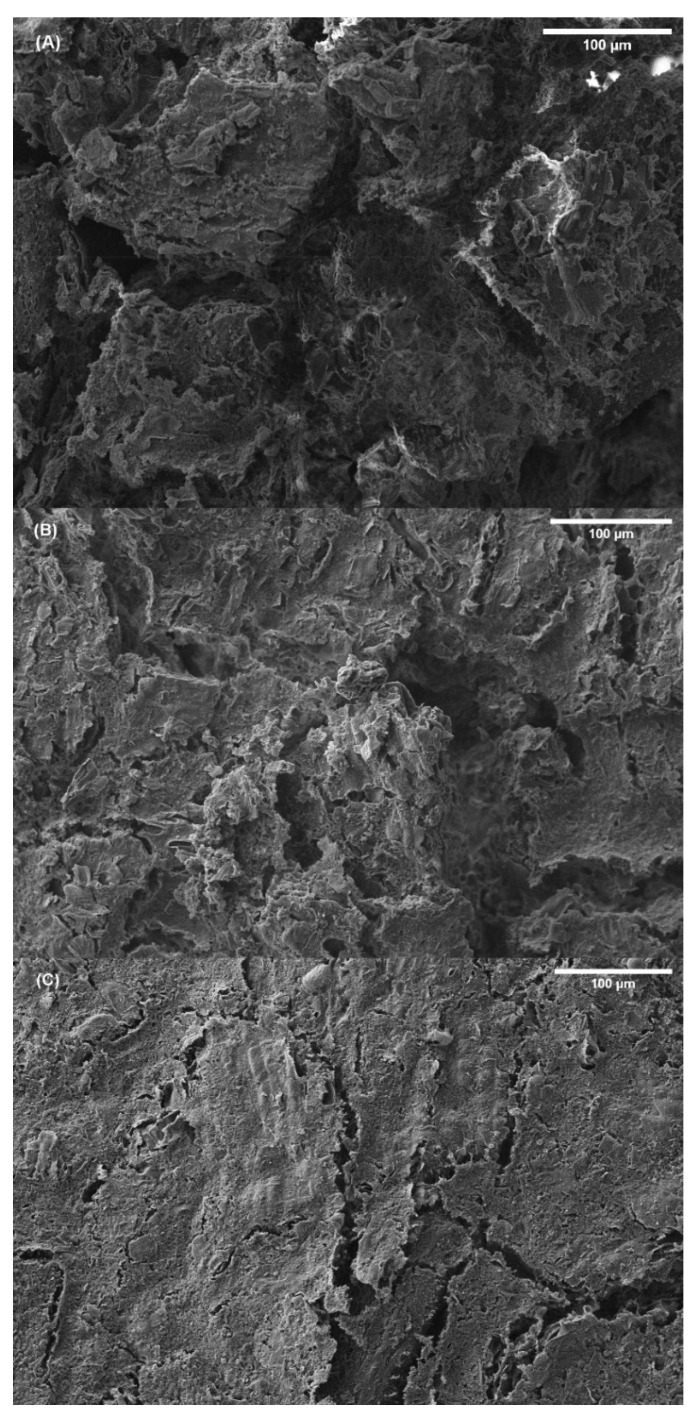
Scanning electron microscopy images of RO/GLY 50/50 (**A**), 60/40 (**B**) and 70/30 (**C**) systems prepared at 120 °C with a magnification of 200×.

Figure 7 shows the images obtained by SEM for each of the bioplastics generated after a freeze drying process after studying the water uptake capacity. By increasing the percentage of seaweed in the formulation, the surface became less porous, which was much more noticeable in the case of the 70/30 system. Furthermore, the lower presence of pores in the structure as the RO/GLY ratio increased was strongly affected by the WUC results (Figure 6): the structure of the 50/50 system was more heterogeneous and showed many structural irregularities and pores through which water can enter, while the 70/30 system showed a lower amount of pores and interstices. Moreover, this also agrees with the viscoelastic and mechanical properties already reported, as the 70/30 system possessed higher viscoelastic moduli, rigidity and resistance supported by a more compact structure, with a lower porosity.

### 3.2. Influence of Molding Temperature in Bioplastic Properties

Bioplastics with a RO/GLY ratio equal to 70/30 were processed at different molding temperatures (T_m_: 90, 120 and 150 °C) to assess their effect on their properties. Previously, some authors have already indicated that T_m_ is the processing parameter that commonly affects the properties of bioplastics to a greater extent when injection molded [23]. 

#### 3.2.1. Dynamic Mechanical Thermal Analysis (DMTA)

**Figure 8:**
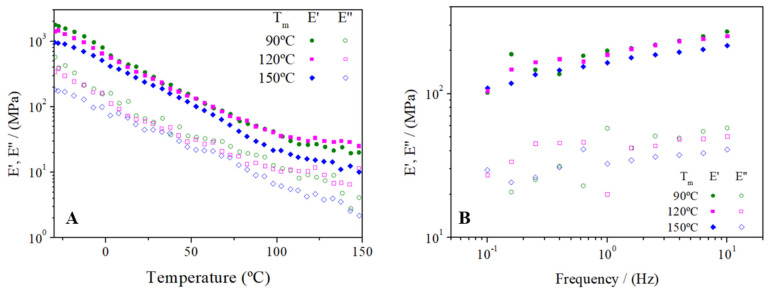
Dependence of viscoelastic moduli on temperature (**A**) and frequency (**B**) for RO/GLY 70/30 bioplastics processed at different molding temperature (90, 120 and 150 °C).

Figure 8 shows the dependence of viscoelastic moduli on temperature (A) and frequency (B) for 70/30 bioplastics molded at different temperatures (90, 120, 150 °C). 

As observed in Figure 4A, all samples processed at different molding temperatures displayed the same qualitative evolution: E’ and E” values, since they decreased when heating the samples and tended to a plateau value around 120 °C. Other authors have previously studied the effect of molding temperature in other bioplastic systems. Thus, Fernández-Espada et al. studied bioplastics based on soy protein molded at 40, 80 and 120 °C, not obtaining significant differences in the evolution of E’ or E” moduli with temperature [23]. However, no great quantitative differences were observed for the 70/30 sample when molded at different temperatures, which would indicate that all molding temperatures used during the holding stage in the injection molding process were high enough to achieve the greatest thermosetting potential of seaweed bioplastics. It should be noted that the sample molded at the highest temperature displayed slightly lower values for both viscoelastic moduli during the whole temperature test, which could be related to the possible degradation of the biomass at 150 °C. 

The frequency sweep tests performed on the 70/30 samples molded at different T_m_ were quite similar (Figure 4B), although the system molded at the highest temperature showed slightly lower E’ and E” values. Moreover, a significant increase was observed in the loss tangents values (tan δ) when increasing T_m_ for the 70/30 system (0.22 ± 0.04, 0.26 ± 0.01, and 0.32 ± 0.01 for 90, 120 and 150 °C, respectively), which implied a loss of the elasticity of the material when molded at higher temperatures. The effects of molding temperature in soy protein-based bioplastics have already been studied by Paetau et al. [38], obtaining similar results. Increasing the temperature favors the crosslinking of the polymer fibers, which makes the material stiffer to some extent. After a certain temperature, proteins are degraded and a decrease in mechanical properties begins to be observed.

#### 3.2.2. Tensile Properties 

**Figure 9:**
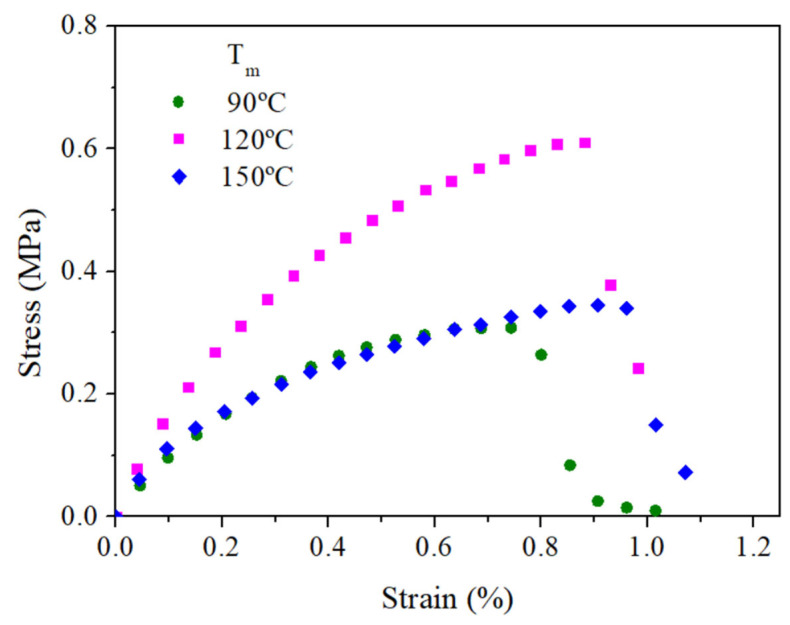
Tensile properties of bioplastics (RO/GLY: 70/30) processed at different molding temperature (90, 120 and 150 °C).

Figure 9 and Table 3 show the stress–strain curves and the main mechanical parameters, respectively, (E, σ_max_, ε_max_) for bioplastics with a RO/GLY ratio of 70/30 processed at different molding temperature (90, 120 and 150 °C). The stress–strain curves of the three systems are similar in shape, with an initial linear elastic behavior, the slope of which decayed slightly until reaching the maximum stress when the material ruptured. Greater stresses are required to deform the sample processed at 120 °C when compared to the rest of mold temperatures, which can resist stresses up to 0.7 MPa; twice the value of that can be applied to the rest of the samples. The initial increase from 90 to 120 °C should be related to a certain strengthening of the sample due to thermally induced interactions (e.g., disulfide bonds), while the decrease in the stress–strain curve from 120 to 150 °C should be associated with the previously commented degradation that negatively affects the mechanical properties [23]. 

#### 3.2.3. Water Uptake Capacity

**Figure 10:**
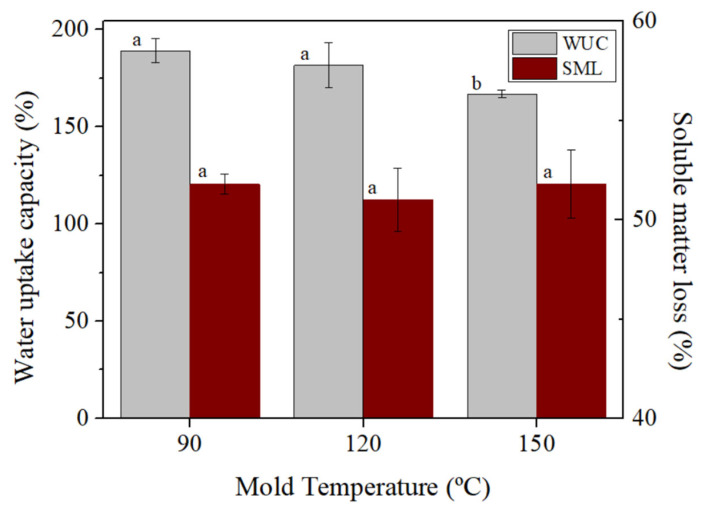
Water uptake capacity and soluble matter loss of the RO/Gly systems 70/30 at different molding temperature (90, 120 and 150 °C). Different letters (a, b) within the same column parameter (WUC or SML) indicate significant differences (*p* < 0.05).

Regarding the WUC, the WUC slightly decreases (Figure 10) the molding temperature increases, being 189.1 ± 6.2% for the system molded at 90 °C, 181.5 ± 11.6% for the 120 °C and 166.8 ± 2.0% for the 150 °C molded system. This effect of temperature on WUC was observed in soy-based bioplastics [23] and was related to the formation of thermally induced interactions that take place during the injection molding. The lowering in WUC is observed from 90 to 150 °C, in spite of the lower viscoelastic moduli and rigidity measured for the sample molded at 150 °C.

No significant differences were observed in the SML values of the systems with T_m_, as all values were around 51.5%, which implies that molding temperature does not exert much influence on soluble matter loss. Different letters within the same column parameter (WUC or SML) indicate significant differences (*p* < 0.05).

#### 3.2.4. Scanning Electron Spectroscopy

**Figure 11:**
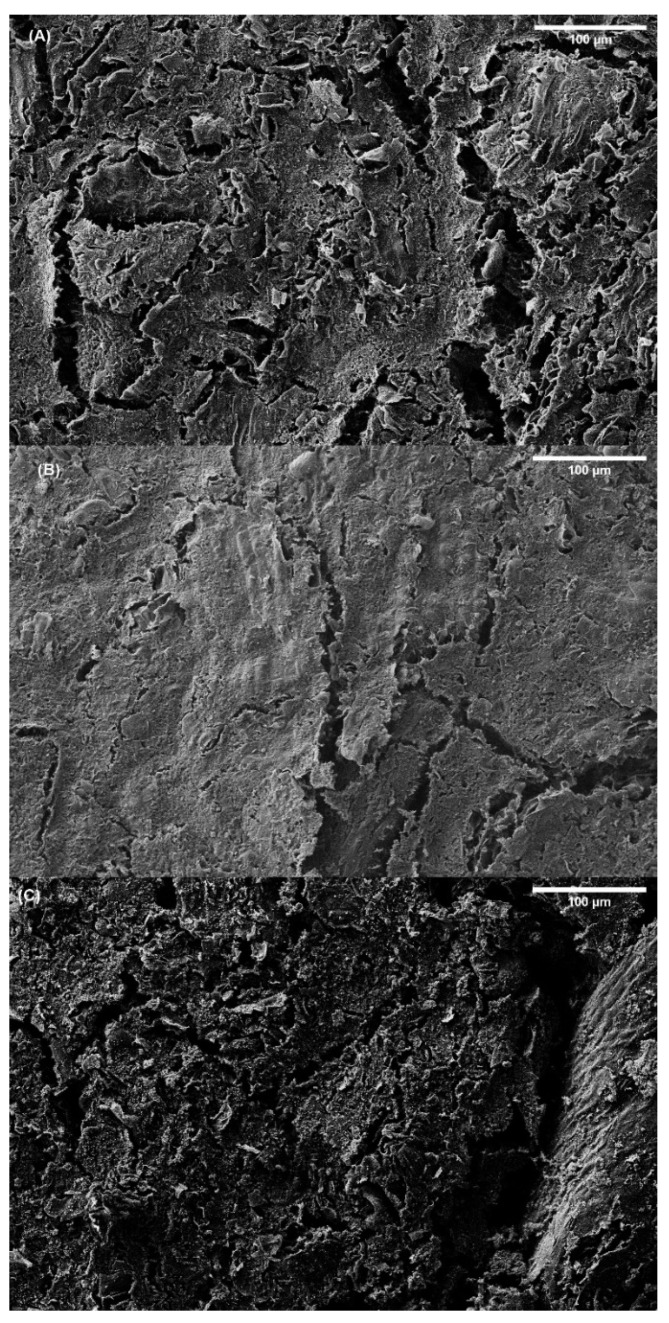
Scanning electron spectroscopy images of RO/GLY 70/30 bioplastics molded at 90 °C (**A**), 120 °C (**B**) and 150 °C (**C**) with a magnification of 200×.

Figure 11 shows the SEM images for bioplastics with a RO/GLY ratio of 70/30 processed at different molding temperatures. Some differences were found between the systems, with the structure of the bioplastic molded at 90 and 150 °C showing greater irregularities and pores than the one molded at 120 °C. These imperfections may be related to a lower degree of cross-linking, resulting in a lower dimensional stability. The smoother and more compact structure observed for the system molded at 120 °C should be related to its lower water uptake capacity and higher viscoelastic properties.

## 4. Conclusions

The invasive seaweed *Rugulopteryx okamurae* can be used to produce bioplastics through injection molding employing glycerol as plasticizer. The higher the content of seaweed, the greater the viscoelastic properties, rigidity and resistance the bioplastics display. Thus, the sample with RO/Gly ratio of 70/30 displayed the higher values of those properties when compared to 50/50 or 60/40 ratios.

Regarding the DMA response, typical thermoplastic behavior was shown, as samples softened when heated until a plateau was achieved at high temperatures. The elastic behavior always prevailed over the viscous one since the storage modulus of every system remained above the loss modulus. The WUC was lowered as the biomass content increased, due to the lower presence of the hydrophilic plasticizer employed. However, some biomass was lost during the immersion of the bioplastic samples, which can be explained by the relatively low mechanical properties of all systems when compared to those obtained from other sources. In relation to this, the greater the mechanical stability of the material, the lower WUC, because the polymers are expected to be more cross-linked and there are greater impediments for water to be in the pores of the structure and bond. This also resulted in a lower SML. The impact of the mold temperature was more noticeable in the tensile test, where the system molded at 120 °C stood out slightly. A slight lowering of water absorption capacity was detected when increasing the mold temperature, although its effect was not very significant when compared to the effect of the formulation (RO/GLY ratio).

The results obtained in the present work are very promising, although further research should be performed to improve the mechanical properties of *Rugulopteryx okamurae* seaweed-based bioplastics. The formulation of composites with other biodegradable polymers and the studies of effects on different processing conditions may be a way to achieve that purpose and to find new utilities.

## Figures and Tables

**Table 1:** Specific Mechanical Energy (SME) involved in the mixing of different RO/GLY systems (t_mix_: 5 min).

RO/GLY	SME (kJ/kg)
50/50	208 ± 37
60/40	328 ± 50
70/30	805 ± 182

**Table 2:** Mechanical parameters (Young’s modulus (E), maximum stress (σ_max_) and maximum strain (ε_max_)) for bioplastics with different RO/GLY ratios (50/50,60/40,70/30). Different letters within a column indicate significant differences (*p* < 0.05).

RO/GLY	E (MPa)	ε_max_ (%)	σ_max_ (MPa)
50/50	0.40 ± 0.14 ^a^	0.88 ± 0.07 ^a^	0.089 ± 0.021 ^a^
60/40	0.70 ± 0.10 ^b^	1.49 ± 0.21 ^b^	0.15 ± 0.03 ^b^
70/30	2.61 ± 0.51 ^c^	0.74 ± 0.12 ^a^	0.68 ± 0.06 ^c^

**Table 3:** Mechanical parameters (Young’s modulus (E), maximum stress (σ_max_) and maximum strain (ε_max_)) of bioplastics (RO/GLY: 70/30): for different molding temperature (90, 120 and 150 °C). Different letters within the same column parameter (WUC or SML) indicates significant differences (*p* < 0.05).

Molding Temperature (°C)	E (MPa)	σ_max_ (%)	ε_max_ (MPa)
90	1.60 ± 0.14 ^a^	0.73 ± 0.002 ^a^	0.33 ± 0.022 ^a^
120	2.61 ± 0.51 ^b^	0.74 ± 0.12 ^a^	0.68 ± 0.057 ^b^
150	1.47 ± 0.17 ^a^	0.69 ± 0.32 ^a^	0.36 ± 0.045 ^a^

## Data Availability

The data presented in this study are available on request from the corresponding author.

## Supplementary Materials

The following are available online at https://www.mdpi.com/article/10.3390/polym14020355/s1, Figure S1: Visual appearance of RO/GLY blends at different ratios (from left to right: 50/50, 60/40, 70/30). Figure S2: Visual appearance of RO/GLY bioplastics at different ratios (from left to right: 50/50, 60/40, 70/30) molded at 120 °C.
