# Supplementary material for: Processing and Characterization of Bioplastics from the Invasive Seaweed Rugulopteryx okamurae
Source: Polymers (Basel). 2022 Jan 17;14(2):355. doi: 10.3390/polym14020355 (PMC8779417; doi:10.3390/polym14020355)
Supplement: Supplementary file 1 [file polymers-14-00355-s001.zip › polymers-1547095-supplementary.pdf]

Supplementary Material

Processing and Characterization of Bioplastics from the Invasive Seaweed *Rugulopteryx okamurae*

Ismael Santana, Manuel Félix, Antonio Guerrero and Carlos Bengoechea *

Higher Polytechnic School, University of Seville; isantana@us.es (I.S.); mfelix@us.es (M.F.); aguerrero@us.es (A.G.)
* Correspondence: cbengoechea@us.es; Tel.: +(34)-954-557-179

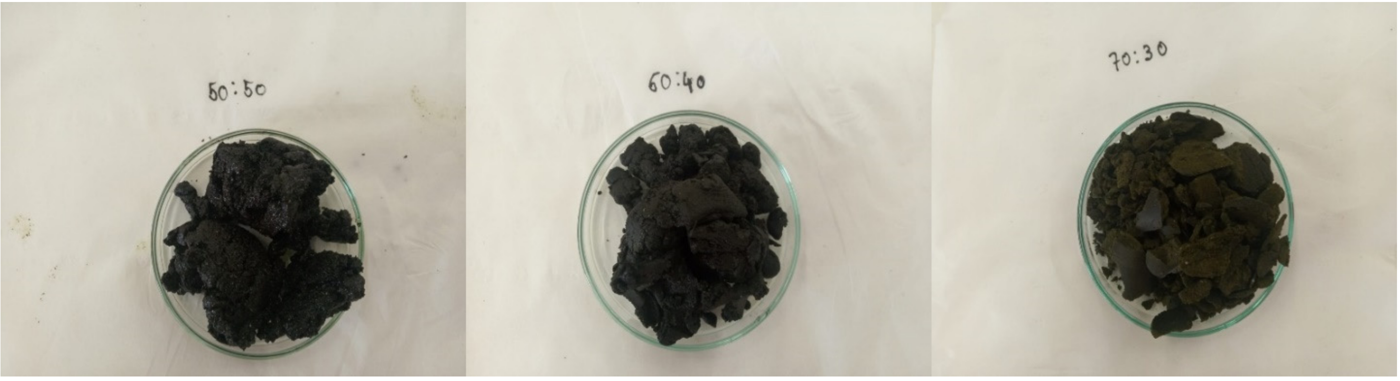

Figure S1. Visual appearance of RO/GLY blends at different ratios (from left to right: 50/50, 60/40, 70/30).

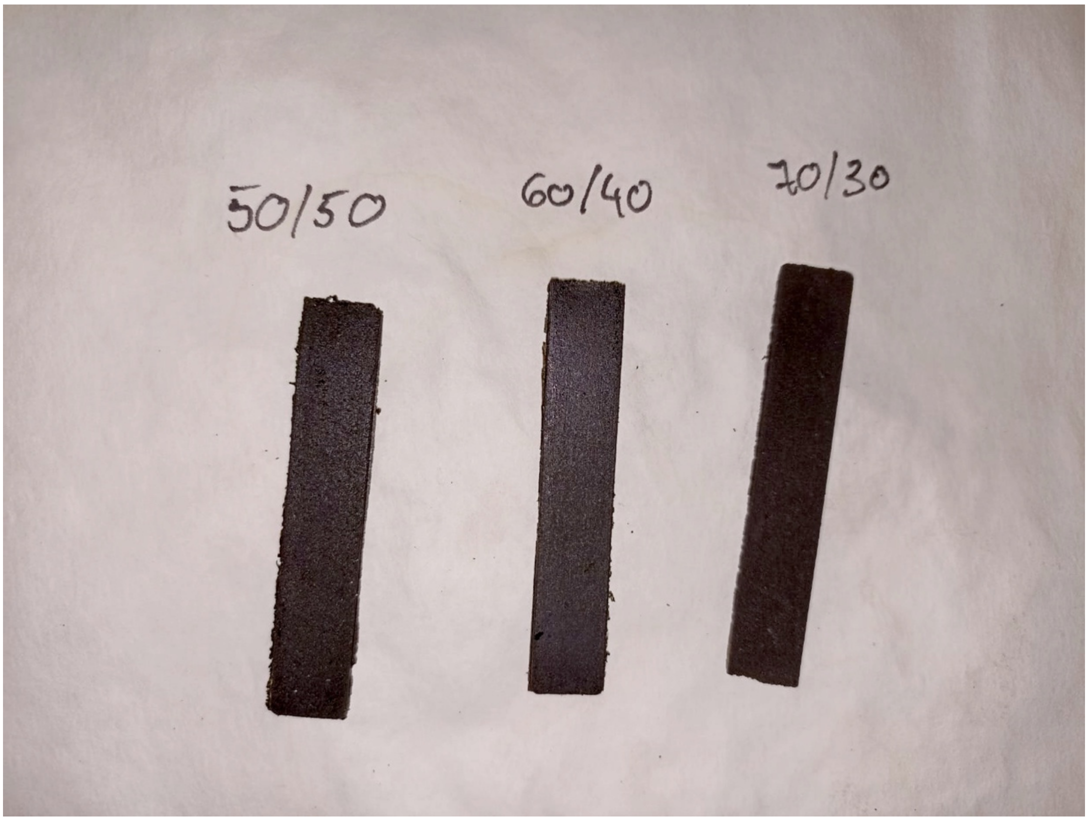

Figure S2. Visual appearance of RO/GLY bioplastics at different ratios (from left to right: 50/50, 60/40, 70/30) molded at 120 °C.
